# Resilience interventions for the family with autistic children: a systematic review

**DOI:** 10.3389/fpsyg.2026.1760817

**Published:** 2026-03-25

**Authors:** Sijia Guo, Huiting Zhang, Jun Wang, Miao Wang

**Affiliations:** 1 College of Public Administration and Humanities, Dalian Maritime University, Dalian, Liaoning, China; 2 Department of Social Work and Social Policy, School of Sociology, Nankai University, Tianjin, China

**Keywords:** autism, family resilience, intervention, social work, systematic review

## Abstract

**Objectives:**

Autism spectrum disorder (ASD) is a lifelong neurodevelopmental condition in which parenting presents considerable challenges. Family resilience is a critical protective factor against adversity. This systematic review aims to identify and evaluate family resilience interventions, based on experimental study designs, for families of children with ASD.

**Methods:**

Following the Preferred Reporting Items for Systematic Reviews and Meta-Analyses (PRISMA) guidelines, we searched 5 electronic databases for peer-reviewed studies published in English prior to July 2025. Eligible studies were empirical studies employing randomized controlled trials (RCT) or quasi-experimental designs; targeted parents/caregivers of children with a clinical diagnosed of ASD; focused on enhancing family resilience; and used validated quantitative measures. Two authors independently screened titles, abstracts, and full texts, extracted study characteristics, and assessed risk of bias using the Cochrane tool. Discrepancies were resolved through discussion or consultation with a third author.

**Results:**

Nine studies were ultimately included. Most interventions were delivered in person, and participants were predominantly mothers. Two primary intervention orientations were identified, family-centered and individual-centered. The majority of studies were conducted in Western countries. Eight studies reported medium to large effect sizes for improvement in family resilience, while 1 study did not report sufficient statistics to calculate effect size.

**Conclusion:**

Despite certain limitations, the findings of this review suggest that future studies should consider cultural differences and contextual adaptation when implementing interventions across diverse cultural settings. The review also highlights the importance of accounting for the differing profiles, needs and preferences of mothers and fathers of children with ASD when designing and facilitating interventions.

## 1 Introduction

Autism spectrum disorder (ASD) is a lifelong neurodevelopmental condition characterized by impairments in social communication and the presence of repetitive or restrictive behavioral patterns. Globally, the prevalence of ASD has continued to rise, with recent estimates suggesting that approximately 1 in 59 children are diagnosed with the disorder (Ghanouni and Hood, 2021), underscoring its increasing public health relevance. Consistent with the social-cognitive difficulties associated with ASD, affected individuals often experience substantial social challenges and functional impairments (Jang et al., 2011). However, the impact of ASD extends beyond the individual level; parents of children with ASD frequently encounter substantial psychological, emotional, and practical difficulties. Children with ASD often exhibit inappropriate, unpredictable, or intense behavioral and emotional reactions, depending on symptom severity (Estes et al., 2013; Rivard et al., 2014), parenting a child with ASD can therefore be a profoundly challenging experience. Furthermore, caring for a child with ASD typically requires considerable time, energy and financial resources, which increases care-giving burden and generates financial strain for parents (Thomas et al., 2016; Vohra et al., 2014).

As a result of these demands, empirical studies have shown that parents of children with ASD report higher levels of stress (Padden and James, 2017), elevated depressive symptoms (Kütük et al., 2021), and more health-related challenges (Ten Hoopen et al., 2020) compared with parents of typically developing children or those with other developmental disabilities. Indeed, parents of children with ASD experience higher stress and lower well-being than parents of children with disorders such as Down syndrome (Picardi et al., 2018).

Despite these challenges, parents vary substantially in how they respond to adversity. Some exhibit adaptive coping and maintain positive psychological functioning, whereas others experience significant emotional distress (Picardi et al., 2018). This variability has prompted increasing interest in identifying protective factors that promote parental well-being. One of the most widely recognized protective factors is resilience, particularly family resilience, which reflects families’ ability to adapt and thrive despite chronic stresses.

Resilience refers to an individual’s capacity to cope with adversity, whereas family resilience extends this concept to the family as a cohesive functional system (Walsh, 2003). Family resilience enables family members to draw upon shared strengths, resources, and adaptive capabilities when confronting challenges. Conceptualizations of family resilience have evolved across three waves of research. The first wave emphasized resilient family traits and resources (McCubbin and McCubbin, 2013). The second wave conceptualized family resilience as a dynamic, multilevel process involving interactions across biological, psychological, relational, and sociocultural systems. The emerging third wave adopts a multidisciplinary perspective, highlighting families’ capacity for positive adaptation and informing the development of prevention and intervention strategies.

Drawing on these three waves of scholarship, Walsh (2003, 2016) proposed a systematic and holistic conceptual framework of family resilience, defining it as the capacity of the family to rebound from crisis and life challenges, and regarding it as a dynamic and interactive process (Walsh, 2002, 2003, n.d). Specifically, this framework encompasses three interrelated domains: belief systems, organizational processes, and communication/problem-solving processes. Belief systems encompass family members’ appraisal of adversity, positive outlook, and transcendence and spirituality. Organizational processes reflect the flexibility and connectedness of the family, as well as its access to resources. Communication/problem-solving processes refer to the degree to which family members engage in clear, open information-sharing and collaborative negotiation in response to crisis and adversity. These domains are mutually interactive and synergistic (Walsh, 2016); for instance, shared meaning-making contributes to communication clarity and emotional sharing, while, reciprocally, communication clarity facilitates shared meaning-making. Taken together, Walsh’s framework offers a comprehensive, systematic and dynamic account of family resilience.

Within families of children with ASD, previous research has shown that family resilience tends to be lower than in families of typically developing children (Al-Jadiri et al., 2021; Schneider et al., 2019). At the same time, family resilience is associated with numerous positive outcomes, including reduced parental depression (Gayatri and Irawaty, 2022), higher psychological well-being and life satisfaction (Ekas et al., 2010), and stronger family relationships (Siman-Tov and Kaniel, 2011). For example, Hsiao (2024) found that family resilience mitigated parental stress and enhanced health-related quality of life among 1,003 U.S. parents. Similarly, Roberts et al. (2017) demonstrated that family resilience contributed to improvements in managing sleep problems among children with ASD. Given the importance of family resilience, it is essential to develop and evaluate interventions designed to strengthen it in families of children diagnosed with ASD. However, few studies have synthesized existing interventions targeting family resilience in this population (Bekhet et al., 2012).

Accordingly, the present systematic review aims to map and examine the current state of family resilience interventions for parents/caregivers of children with ASD, grounded in Walsh’s family resilience framework. Given that family resilience is a multi-systematic concept, we consider a range of service recipients, including mothers, fathers, and other caregivers of children with ASD. Since family resilience encompasses interactive dimensions, we include intervention studies targeting any domain of family resilience – namely, belief systems, organizational processes, and communication/problem-solving processes, or family resilience as an integrated outcome across various approaches and therapeutic modalities.

Specifically, this review aims to: (1) identify the formats and target populations of existing interventions; (2) characterize the intervention approaches and specific therapeutic techniques employed; and (3) evaluate the effect sizes of these interventions on dimensional family resilience outcomes.

## 2 Methods

### 2.1 Search strategy

**FIGURE 1:**
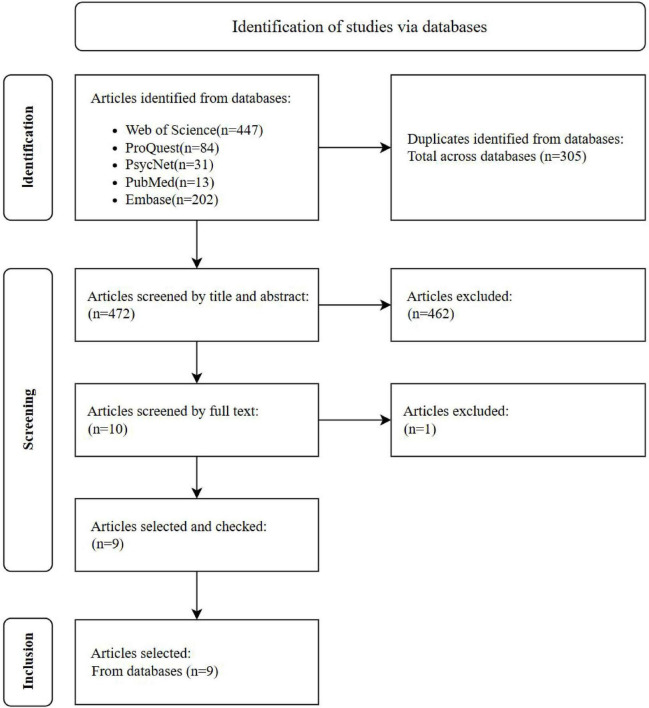
Search and selection process to identify family resilience intervention articles on the ASD families.

This systematic review was conducted in accordance with the Preferred Reporting Items for Systematic Reviews and Meta-Analyses (PRISMA) guidelines (Moher et al., 2009; Page et al., 2020). Figure 1 provides an overview of the search and study selection process. We searched multiple electronic databases, including Web of Science, PubMed, PsycNet, ProQuest, and Embase, for relevant studies published prior to July 2025.

The search syntax applied across all databases was as follows: (Autism OR Autis* disorder) AND (family OR carer* OR caregiver*) AND (parent* resilienc* OR famil* resilienc*) AND (intervention OR treatment OR program OR outcome OR efficacy OR effectiveness OR experiment OR trial). This syntax integrates keywords corresponding to three core domains: the research focus (family resilience), the target population (parents or caregivers of children with ASD), and the research design (intervention studies).

### 2.2 Eligibility criteria

In line with the aims of this systematic review, a family resilience intervention was defined as any structured program, treatment or intervention that directly or indirectly targets change in any domain of family resilience, including belief systems, organizational processes and communication/problem-solving processes, or that measures integrated family resilience as an outcome. Studies were included if they: (a) evaluated interventions designed to improve family resilience outcomes among parents or caregivers of children with ASD; (b) employed an empirical methodology with randomized controlled trials (RCT) or quasi-experimental designs; (c) directly targeted family resilience or indirectly targeted variables theoretically linked to resilience; (d) used validated measurement instruments and reported quantitative results; (e) involved parents/caregivers of clinically diagnosed autistic children; (f) were published in peer-reviewed journals; and (g) were published in English.

Studies were excluded if they were non-intervention in designs, or took the form of dissertations, theses, reviews, conceptual papers, as well as any studies for which full-text access was unavailable.

### 2.3 Study selection

Two authors independently screened all titles and abstracts, with discrepancies resolved through discussion, and unresolved conflicts adjudicated by a third reviewer. Full texts of eligible studies were subsequently retrieved. Given the large volume of full-text articles, two authors independently reviewed a random subset of 100 full texts, achieving 100% inter-rater agreement. On the basis of this agreement, the first author reviewed the remaining full-text articles. Studies meeting all inclusion criteria were retained for analysis.

### 2.4 Data extraction

For each included study, data were extracted on publication year, country, intervention duration and sample size. Additional details were recorded regarding intervention format (e.g., group-based), target population (e.g., mothers), intervention focus, and measured outcomes.

### 2.5 Assessment of risk of bias

Risk of bias was evaluated using the Cochrane Risk of Bias tool (Cumpston et al., 2019), across seven domains: random sequence generation, allocation concealment, blinding of participants and personnel, blinding of outcome assessment, incomplete outcome data, selective outcome reporting, and other potential source of biases. Two authors independently assessed each study, with disagreements resolved through discussion. Each domain was classified as low, unclear, or high risk of bias in accordance with Cochrane guidelines (Cumpston et al., 2019).

## 3 Results

### 3.1 Article review process

The PRISMA flow diagram (Figure 1) summarizes the search process. The initial search yielded 777 records. After screening titles and abstracts, 767 articles were excluded, leaving 10 articles for full-text assessment. One study was excluded at the full-text stage for not meeting the inclusion criteria. Ultimately, 9 studies were included in the final review.

### 3.2 Characteristics of included studies

Table 1 summarizes the characteristics of the 9 included studies.

**TABLE 1:** Characteristics of included interventions on family resilience of the ASD families.

References	Participants	Duration	Intervention design	Sample size	Country
Kuhlthau et al., 2020	Parents of children with ASD, with 49 mothers (accounting for 96.1%)	8 weeks, 90 min per session, no follow-ups	Pilot RCT	Treatment: *N* = 20; Comparison: *N* = 22	The United States
Pandya, 2021	Mothers of children with ASD (accounting for 100%)	50 weeks, no follow-ups	RCT	Treatment: *N* = 68; Comparison: *N* = 46	Not reported
Stoliaryk and Semigina, 2022	Parents or caregivers of children with ASD, with 52 mothers (accounting for 58%)	3 months, 150 min per session, no follow-ups	Pilot study	Treatment: *N* = 30 Comparison: *N* = 60	Ukraine
Schwartzman et al., 2022	Parents of children with ASD, with 26 mothers (accounting for 76.5%)	8 weeks, 90 min per session, with follow-ups in 2 months	RCT	Treatment: *N* = 17, Comparison: *N* = 17	The United States
Wachspress et al., 2024	Parents of children with ASD (with 4 couples and six mothers, mothers accounting for 71.4%)	12 weeks, 90 min per session, with follow-ups in 3 months	Pilot study	Treatment: *N* = 14	Israel
Bravo-Benítez et al., 2024	Family caregivers of children with ASD, with 24 mothers (accounting for 85.7%)	10 weeks, 90 min per session, no follow-ups	RCT	Treatment: *N* = 14; Comparison: *N* = 14	Spain
Sabanciogullari and Yıldırım, 2025	Parents of children with ASD, with 14 mothers (accounting for 46.7%)	10 weeks, 120 min per session, no follow-ups	Quasi-experimental	Treatment: *N* = 16; Comparison: *N* = 14	Turkey
Wachspress et al., 2025	Parents of children with ASD (with 12 couples, 20 mothers and 4 fathers, mothers account for 66.7%)	11 weeks, 90 min per session, with follow-ups in 3 months	RCT	Treatment: *N* = 30 Comparison: *N* = 18	Israel
Cengiz and Kılıç, 2025	Parents of children with ASD, with 60 mothers (accounting for 88.2%)	10 weeks, 45 min per session, no follow-ups	RCT	Treatment: *N* = 34, Comparison: *N* = 34	Turkey

Most participants were mothers of children with ASD, although some studies included other primary caregivers. Intervention duration ranged from 8 to 50 weeks, and only 3 studies included follow-up assessments (Schwartzman et al., 2022; Wachspress et al., 2024, 2025). Sample sizes ranged from 14 to 34 participants in treatment groups and from 14 to 60 participants in control groups, 1 study included a treatment group only. Furthermore, most of the included studies were conducted in Western countries, including the United States, Ukraine, Spain and Turkey. Only 1 study was conducted in Asia - specially in Israel, and 1 study did not report the study location.

### 3.3 Risk of bias of included studies

Only 1 study (Pandya, 2021) reported blinding of both participants and personnel. Regarding outcome assessment blinding, only Cengiz and Kılıç (2025) explicitly reported adequate procedures. Based on the Cochrane criteria, 6 studies were rated as having an unclear risk of bias (Bravo-Benítez et al., 2024; Kuhlthau et al., 2020; Sabanciogullari and Yıldırım, 2025; Schwartzman et al., 2022; Stoliaryk and Semigina, 2022; Wachspress et al., 2025). Two studies demonstrated a low risk of bias (Pandya, 2021; Cengiz and Kılıç, 2025). Only 1 study showed a high risk of bias due to inadequate randomization procedures (Wachspress et al., 2024). Table 2 presents the full risk-of-bias assessment.

**TABLE 2:** Risk of bias assessment of included studies to enhance family resilience of the ASD families.

Numbers	References	Random sequence generation	Allocation concealment	Blinding of participants and personnel	Blinding of outcome assessment	Incomplete outcome data	Selective outcome reporting	Other bias	Summary
1	Kuhlthau et al., 2020	Yes (use a random plan generator)	No information	No	No information	No information	Included all expected outcomes	No information	Unclear risk
2	Pandya, 2021	Yes (use computer generated random numbers)	No information	Yes	No information	No information	Included all expected outcomes	No information	Low risk
3	Stoliaryk and Semigina, 2022	No information	No information	No information	No information	No information	Included all expected outcomes	No information	Unclear risk
4	Schwartzman et al., 2022	Yes (use electronic random number generation)	No	No information	No information	No information	Included all expected outcomes	No information	Unclear risk
5	Wachspress et al., 2024	No	No information	No information	No information	No information	Included all expected outcomes	No information	High risk
6	Bravo-Benítez et al., 2024	Method of randomization not mentioned	No information	No information	No information	No information	Included all expected outcomes	No information	Unclear risk
7	Sabanciogullari and Yıldırım, 2025	Method of randomization not mentioned	No information	No information	No information	No information	Included all expected outcomes	No information	Unclear risk
8	Wachspress et al., 2025	Yes (use WINPEPI software with minimization assignment)	No information	No information	No information	No information	Included all expected outcomes	No information	Unclear risk
9	Cengiz and Kılıç, 2025	Yes (use a computer-generated randomization code)	No information	No	Yes	No information	Included all expected outcomes	No information	Low risk
Note:									
Low risk of bias		Unclear risk of bias	High risk of bias						

### 3.4 The major information of intervention

This section presents the main findings of the included studies, focusing on the intervention modality, format, participant characteristics, and effect sizes (see Table 3).

**TABLE 3:** Summary of intervention strategies and findings of included studies on ASD families.

References	Intervention orientation	Specific intervention therapy	Targeted domain of Family resilience	Delivery method	Outcome measurement	Intervention outcomes	Effect size
Wachspress et al., 2024	Family-centered	Occupational therapy	Organizational processes and communication/problem-solving processes	Offline	Parenting Questionnaire (APQ)	Significant improvements in performance scores for both mothers and adolescents and significant improvement in parent resilience.	Large
Wachspress et al., 2025	Family-centered	Occupational therapy	Organizational processes and communication/problem-solving processes	Offline	APQ	Significant improvement in parents’ psychological resilience, parents’ self-determination, parents’ occupational performance and satisfaction, autistic adolescents’ occupational performance and satisfaction.	Large
Stoliaryk and Semigina, 2022	Family-centered	Strengths-based intervention	Organizational processes and communication/problem-solving processes	Offline	APQ	Significant improvement in parents’ psychological resilience, parents’ marital relationship quality and parents’ parental competence.	Not reported
Sabanciogullari and Yıldırım, 2025	Family-centered	McMaster model of family functioning	Organizational processes and communication/problem-solving processes	Offline	Resilience Scale for Adults (RSA)	Significant improvement in parents’ psychological resilience.	Medium
Pandya, 2021	Individual-centered	Spirituality-based intervention	Belief systems	Online	Parenting Resilience Elements Questionnaire (PREQ)	Significant reduction in parenting stress, significant improvement in parental self-efficacy, maternal confidence and resilience.	Large
Bravo-Benítez et al., 2024	Individual-centered	Grief therapy	Belief systems	Offline	Connor-Davidson Resilience Scale-10 (CD-RISC-10)	Significant reduction in caregiver burden; significant increase in resilience and positive aspects of care-giving.	Medium-to-large
Kuhlthau et al., 2020	Individual-centered	Mind-body training, cognitive behavioral therapy, positive psychology approaches	Organizational processes and communication/problem-solving processes	Online	Current Experience Scale (CES)	Significant improvements in resiliency and stress reactivity/coping; greater improvements in mindfulness with medium-to-large effect sizes.	Medium-to-large
Schwartzman et al., 2022	Individual-centered	Cognitive behavioral therapy, mindfulness-based stress reduction, acceptance and commitment therapy	Organizational processes and communication/problem-solving processes	Offline	Connor-Davidson Resilience Scale-25 (CD-RISC-25)	Significant improvement in parents’ psychological resilience, and mindfulness; significant reduction in parenting stress.	Large
Cengiz and Kılıç, 2025	Individual-centered	Mindfulness-based compassionate living	Belief systems	Hybrid	RSA	Significant improvement in parents’ psychological resilience, self-compassion and mindfulness; significant reduction in parental stress.	Large

#### 3.4.1 The implementation methods and participants

Across all included studies, offline in-person delivery was the predominant intervention format, employed by 6 studies. In contrast, 2 studies implemented fully online interventions (Kuhlthau et al., 2020; Pandya, 2021), and 1 study used a hybrid approach combining online and offline elements (Cengiz and Kılıç, 2025). Although online intervention shared a consistent advantage in terms of accessibility and cost-effectiveness, the 3 online or hybrid studies employed different platforms depending on their intervention objectives. For instance, Pandya (2021) utilized WhatsApp, which enables participants to share images and documents, and exchange text messages in real time. By contrast, the remaining 2 studies used video conferencing tools, such as Zoom, to conduct intervention sessions (Cengiz and Kılıç, 2025; Kuhlthau et al., 2020).

Regarding participant characteristics, a notable degree of consistency was observed across studies, with mothers comprising the majority of intervention participants. Most included studies aimed to recruit the primary caregivers of children with ASD; an exception was Pandya (2021), which exclusively recruited mothers due to the nature of intervention design.

#### 3.4.2 The orientations and specific therapies

Among the selected studies, two orientations were identified: family-centered and individual-centered. These orientations differ in their conceptual focus and employ distinct strategies when delivering intervention.

Family-centered interventions emphasize a systemic and ecological view of family resilience, in which programs target the family as a whole. The included studies within this orientation addressed two domains of family resilience that are organizational processes and communicational/problem-solving processes by enhancing parenting performance, modifying family relationships, and strengthening family functioning. Although different therapeutic approaches were applied, all adhered to an ecological framework. For instance, Wachspress et al. (2024, 2025) applied Occupational Therapy to foster a collaborative partnership between parents and their autistic children, with the aim of promoting parental competence and adolescents’ participation in daily activities. Sabanciogullari and Yıldırım (2025) implemented a Family Counseling Program to provide psychosocial support related to family relationships, care-giving responsibilities and problem-solving strategies for parents of children with ASD. Stoliaryk and Semigina (2022) employed a strength-based intervention focusing on expanding resources for families of children with ASD, reorganizing family systems, and restructuring family values to enhance overall quality of life.

Individual-centered interventions, by contrast, emphasize a strengths-based perspective of family resilience, with the primary focus on individual family members. The findings indicate that these studies prioritized the belief systems domain of family resilience. Notably, individual-centered interventions commonly incorporated multiple collaborative therapeutic modalities. For instance, Cengiz and Kılıç (2025) employed the Mindfulness-Based Compassionate Living program, which integrates mindfulness and compassion-based approaches. Schwartzman et al. (2022) combined Cognitive Behavioral Therapy, Mindfulness-Based Stress Reduction, and Acceptance and Commitment Therapy to develop the AMOR (Acceptance, Mindfulness, Optimism, Resilience) program. Kuhlthau et al. (2020) developed the Stress Management and Resiliency Training-Relaxation Response Resiliency Program by integrating relaxation elicitation techniques, stress management techniques and positive psychology principles.

#### 3.4.3 The outcome and effect size of interventions

Family-centered interventions prioritized outcomes related to family functioning, family communication, family relationships, and parental occupational performance. For instance, Wachspress et al. (2024, 2025) used the Canadian Occupational Performance Scale measure to track changes in participants’ self-perceived occupational performance and satisfaction. Sabanciogullari and Yıldırım (2025) administered the Family Assessment Device Scale to measure family functioning across dimensions including problem-solving, communication, roles, affective responsiveness, affective involvement, behavioral control and general functioning.

Individual-centered interventions, in contrast, focused on outcomes related to emotional status, self-awareness, mental health and psychological flexibility. Notably, all individual-centered studies measured changes in stress levels. In addition, both studies from Bravo-Benítez et al. (2024) and Schwartzman et al. (2022) used the Acceptance and Action Questionnaire to assess personal psychological flexibility. Two studies examined mindfulness outcomes (Cengiz and Kılıç, 2025; Kuhlthau et al., 2020), and 2 studies assessed positive affect more broadly (Bravo-Benítez et al., 2024; Cengiz and Kılıç, 2025). The variation in other measured outcomes reflects the tailored nature of each intervention orientation.

Regarding intervention effectiveness on resilience, most studies reported medium to large effect sizes, indicating that the included interventions were generally effective in enhancing dimensional or integrated family resilience. Stoliaryk and Semigina (2022) did not report sufficient statistical data, precluding the calculation of an effect size.

## 4 Discussion

Parents/caregivers of children with ASD experience substantial stress and emotional challenges. Family resilience is a key protective factor that enables families to adapt to adversity. The interventions reviewed here offer insights into approaches that may strengthen family or parental resilience.

### 4.1 The implementation method and participants

Given the intensive time and energy required to care for children with ASD, intervention delivery format (e.g., in-person vs. online) is likely to influence feasibility and acceptability. Empirical studies indicated the strengths of in-person intervention, including rapid trust-building, enhanced social connectedness, and a more natural interpersonal climate (Sharaievska and Burk, 2018). However, there are several limitations of in-person intervention, for instance time constrains the participation rate of parents/caregivers of children with ASD. Compared with in-person format, online intervention breaks the constrains, reduces financial burden, and offers flexibility and accessibility for the families with ASD children, which improves the parents’ participants and fidelity (Bonis, 2016; Fox et al., 2017; Galpin et al., 2018). Nevertheless, this kind of intervention also exists some disadvantages, such as technical or practical issues, or distractions from children at home (Lodder et al., 2020; Banbury et al., 2014). With regard to the strengths and limitations of in-person and online formats, some studies have tried to explore the effectiveness of the hybrid-format intervention. For instance, Lodder et al. (2020) conducted a blended format psychosocial group support intervention to improve the mental health of parents/caregivers of ASD children, and the result indicated that a hybrid format works best. Herein, it is necessary to consider the caregivers’ needs, the target of intervention and contextual constrains when designing the format of intervention in the future in order to enhance the feasibility, acceptability and effectiveness of intervention.

Parents/caregivers of children often report high level of stress, heavy caregiving burden, social isolation and negative maternal relationship (Iacob et al., 2020; Lee, 2013), which means they need both internal and external resources to cope effectively with chronic stress and adversity. This study finds that mothers with ASD children constitute the majority of intervention participants, which is consistent with the empirical studies that mothers are assumed as the primary caregiver who undertake care-giving responsibilities (Lee, 2013). Besides, the study from Ozturk et al. (2014) found that mothers of ASD children actually engage in more social behavior with their children than fathers, and mothers tend to take on more parenting responsibilities. Moreover, some of empirical studies also pointed that mothers of ASD children experience more depression and stress than fathers, while few studies did not show any significant differences between mothers and fathers in terms of stress or depression (Ozturk et al., 2014; Davis and Carter, 2008; Hastings, 2003). Although some studies compared the similarities and differences between mothers and fathers of ASD children, their profiles are not clear enough that needs further exploration (Flippin and Crais, 2011). What is more, it is essential to consider the different needs or preferences between male and female. For female, they tend to gain more from spiritual techniques, such as reducing rumination that is the natural behavioral response of female to cope with stressful events (Rojiani et al., 2017). However, male may need externalizing activities to reduce stress in daily life (Johnson and Whisman, 2013). Therefore, for the future study, it is vital to explore and to clarify the needs of caregivers before conducting interventions.

### 4.2 The orientation and specific therapy of intervention

The two orientations of interventions, family-centered and individual-centered, reflect the multidimensional nature of family resilience. As mentioned above, the concept of family resilience involves the dynamic interactions among family members, family subsystems and sociocultural systems (Walsh, 2003). Meanwhile, family resilience consists of three domains that are mutually interactive (Walsh, 2016). Accordingly, researchers focus on diversified facets to improve family resilience, which are family-centered and individual-centered respectively. Followed family-centered approach, these interventions uphold ecological and systematic perspective focusing on parental competence, family relationship and family functioning, while the interventions guided by individual-centered highlight the aspect of belief systems by modifying personal cognition, improving individual mental health and enhancing self-compassion and self-awareness. Although these interventions emphasize varied aspects, the results from them show that parental psychological health state or capability becomes better, the family functioning and the well-being of family would be better, and *vice versa*. This finding indicates the interactive processes of family resilience. From this perspective, it could be told that we can design intervention from a holistic perspective by targeting at different domains to improve family resilience, as well as we can only focus on one domain of family resilience to design the intervention.

Besides, it should be noted that the core processes of family resilience could be expressed in varied ways, which is related to cultural norms and family preferences (Walsh, 2016). Therefore, culture is an important indicator to be considered during conducting intervention, as individuals are embedded within the surrounding social context and culture that shape and influence individuals’ interactions, experiences, and beliefs, etc., (Pudjiati et al., 2021). For instance, the study from Pandya (2021) emphasized the importance of culture, as spirituality appeals more to those who can find it culturally familiar (Woods-Giscombé and Gaylord, 2014). Additionally, different culture emphasizes different dimensions of family resilience (Gunnestad, 2006). For instance, collectivism cultures often emphasize interdependence, filial obligations, and shared problem-solving, which can strengthen relational cohesion and communal support as central components of family resilience. In contrast, families in more individualistic cultural contexts may rely more on personal autonomy, emotional expressiveness, and individual coping strategies, illustrating distinct pathways through which resilience is enacted. These cultural variations indicate that family resilience is not a universal or fixed construct but a dynamic process influenced by sociocultural contexts (Roman et al., 2025). Understanding these cultural nuances is essential for both research and practice.

### 4.3 The outcome and effect size of interventions

Across studies, interventions improved resilience and yielded positive secondary outcomes such as increased parental competence, improved occupational performance, and reduced care-giving burden. These findings align with previous research demonstrating that family resilience enhances parental well-being and family functioning (Ekas et al., 2010; Gayatri and Irawaty, 2022; Siman-Tov and Kaniel, 2011).

Several factors may explain variability in effect sizes. Longer intervention duration may produce stronger effects, possibly because participants need sufficient time to practice and internalize new skills (Ridderinkhof et al., 2018). In addition, interventions with greater interpersonal engagement, such as one-to-one guidance or interactive group components, also tend to yield larger effects (Vadasy et al., 1997).

### 4.4 Limitations and future research directions

This review has several limitations. Although we conducted a systematic search, additional databases and search terms may have identified further eligible studies. Our analysis relied solely on published studies, which may exclude relevant but unpublished interventions. Additionally, some included studies lack full information or data. Meanwhile, some studies are pilot studies that only illustrate the acceptability and feasible of the intervention, which needs further demonstration by conducting a randomized controlled trial. Furthermore, the included studies did not design a comprehensive intervention by integrating individual, family and community levels. The future studies are suggested to report adequate and full information about the intervention, as well as designing a comprehensive and normative intervention for the family of children with ASD.

To conclude, this systematic review analyzed 9 included studies and found that offline format are the major form of current intervention, though some adopt online format or hybrid format; mothers accounts for the largest proportion of participants in the intervention; there are two orientations with multiple therapies among the included studies; most of interventions show a medium to large effect size. These findings have valuable implications for future study.
